# Supplementary material for: Modeling glioblastoma heterogeneity as a dynamic network of cell states
Source: Mol Syst Biol. 2021 Sep 16;17(9):e10105. doi: 10.15252/msb.202010105 (PMC8444284; doi:10.15252/msb.202010105)
Supplement: Supplementary file 6 — Source Data for Figure 5 [file MSB-17-e10105-s004.zip › Figure5A_sourcedata/GSEA_3017/nosigdb_stateE.GseaPreranked.1622795031093/NB_KI_CLUSTER18.html]

Details for gene set NB\_KI\_CLUSTER18[GSEA]

|  || Dataset | state63017 |
| Phenotype | NoPhenotypeAvailable |
| Upregulated in class | na\_pos |
| GeneSet | NB\_KI\_CLUSTER18 |
| Enrichment Score (ES) | 0.80205715 |
| Normalized Enrichment Score (NES) | 3.8360639 |
| Nominal p-value | 0.0 |
| FDR q-value | 0.0 |
| FWER p-Value | 0.0 |
Table: GSEA Results Summary

  

Fig 1: Enrichment plot: NB\_KI\_CLUSTER18      
 Profile of the Running ES Score & Positions of GeneSet Members on the Rank Ordered List

  

| PROBE | GENE SYMBOL | GENE\_TITLE | RANK IN GENE LIST | RANK METRIC SCORE | RUNNING ES | CORE ENRICHMENT || 1 | CYR61 |  |  | 3 | 1.169 | 0.0873 | Yes |
| 2 | IGFBP5 |  |  | 4 | 1.015 | 0.1658 | Yes |
| 3 | CCL2 |  |  | 5 | 0.975 | 0.2413 | Yes |
| 4 | ID3 |  |  | 6 | 0.972 | 0.3165 | Yes |
| 5 | SPARCL1 |  |  | 7 | 0.963 | 0.3910 | Yes |
| 6 | CEBPD |  |  | 15 | 0.791 | 0.4447 | Yes |
| 7 | PRSS23 |  |  | 21 | 0.722 | 0.4952 | Yes |
| 8 | CYTOR |  |  | 23 | 0.704 | 0.5486 | Yes |
| 9 | LGALS3 |  |  | 25 | 0.697 | 0.6015 | Yes |
| 10 | IFITM3 |  |  | 26 | 0.682 | 0.6543 | Yes |
| 11 | CALD1 |  |  | 41 | 0.580 | 0.6841 | Yes |
| 12 | LHFPL6 |  |  | 71 | 0.451 | 0.6877 | Yes |
| 13 | CST3 |  |  | 86 | 0.425 | 0.7054 | Yes |
| 14 | CAVIN1 |  |  | 100 | 0.399 | 0.7223 | Yes |
| 15 | TIMP3 |  |  | 106 | 0.391 | 0.7471 | Yes |
| 16 | CD99 |  |  | 107 | 0.389 | 0.7772 | Yes |
| 17 | LGALS1 |  |  | 113 | 0.368 | 0.8003 | Yes |
| 18 | SOCS3 |  |  | 141 | 0.342 | 0.7976 | Yes |
| 19 | FILIP1L |  |  | 167 | 0.320 | 0.7954 | Yes |
| 20 | A2M |  |  | 184 | 0.309 | 0.8021 | Yes |
| 21 | ZFP36L1 |  |  | 287 | -0.255 | 0.7117 | No |
Table: GSEA details [plain text format]

  

Fig 2: NB\_KI\_CLUSTER18: Random ES distribution      
 Gene set null distribution of ES for **NB\_KI\_CLUSTER18**
